# Monoclonal antibodies in the management of asthma: Dead ends, current status and future perspectives

**DOI:** 10.3389/fimmu.2022.983852

**Published:** 2022-12-06

**Authors:** Grzegorz Kardas, Michał Panek, Piotr Kuna, Piotr Damiański, Maciej Kupczyk

**Affiliations:** Clinic of Internal Medicine, Asthma and Allergy, Medical University of Lodz, Łódź, Poland

**Keywords:** asthma, severe asthma, monoclonal antibodies, tezepelumab, dupilumab, benralizumab, mepolizumab, omalizumab

## Abstract

Patients with moderate-to-severe asthma may now be treated using a variety of monoclonal antibodies that target key inflammatory cytokines involved in disease pathogenesis. Existing clinical data on anti-IgE, anti-IL-5 and other immunological pathways indicate these therapies to offer reduced exacerbation rates, improved lung function, greater asthma control and better quality of life. However, as several patients still do not achieve satisfactory clinical response with the antibodies available, many more biologics, aiming different immunological pathways, are under evaluation. This review summarizes recent data on existing and potential monoclonal antibodies in asthma. Recent advances have resulted in the registration of a new antibody targeting TSLP (tezepelumab), with others being under development. Some of the researched monoclonal antibodies (e.g. anti-IL-13 tralokinumab and lebrikizumab or anti-IL-17A secukinumab) have shown optimistic results in preliminary research; however, these have been discontinued in asthma clinical research. In addition, as available monoclonal antibody treatments have shown little benefit among patients with T_2_-low asthma, research continues in this area, with several antibodies in development. This article summarizes the available pre-clinical and clinical data on new and emerging drugs for treating severe asthma, discusses discontinued treatments and outlines future directions in this area.

## Introduction

Asthma is a chronic, heterogeneous and inflammatory respiratory condition characterized by shortness of breath, cough, wheezing, and chest tightness. It belongs to the group of obstructive diseases characterized by variable airflow limitation (1). Asthma occurs in several phenotypes that vary in their pathogenesis, and the intensity and frequency of symptoms and exacerbations (2). Currently, its prevalence is estimated to reach 1-18% depending on the country studied (1, 3, 4). The disease affects all age groups, with new cases diagnosed predominantly in children aged 0-9 [early-onset asthma, usually atopic (5)] and in adults aged 40-49 [late-onset asthma, often eosinophilic phenotype (6)]. It is important to note that, as is the case for other allergic diseases, its global prevalence is increasing (4, 7, 8), which has been attributed to various factors such as air pollution, antibiotic misuse, viral infections and a high-hygiene lifestyle (9, 10).

The term *asthma* is currently considered an umbrella term that encompasses several clinical and pathophysiological variants. The main axis of division refers to the type of inflammation, i.e. type 2 inflammation and non-type 2 inflammation. Furthermore, asthma phenotypes are considered as either eosinophilic or non-eosinophilic, with blood eosinophil count considered a major, yet controversial, phenotype-distinguishing biomarker (11). Most patients present a T_h_2-predominant allergic phenotype asthma, which develops on basis of atopy triggered by inhaled allergens, e.g. house dust mite, grass pollens, trees or pets (6). Apart from the classical, early-onset allergic asthma, late-onset eosinophilic asthma is also under intensive study (12), as are other various asthma phenotypes, including obesity-associated asthma, neutrophilic asthma and very-late onset asthma. Asthma pathogenesis is strongly influenced by a number of mediators of inflammation, such as IgE, IL-3, IL-4, IL-5, IL-9, IL-13, IL-33 and TSLP, with many more being discovered (13).

### Current clinical options for the treatment of severe asthma

In clinical practice, three levels of asthma severity are distinguished (mild, moderate and severe), with treatment being based on the five Global Initiative for Asthma (GINA) steps (1). The most severe cases, in which asthma control is not reached despite using high doses of inhaled corticosteroids, may be qualified for GINA step 5 biological treatment with monoclonal antibodies that are targeting key asthma mediators.

According to current epidemiological data, 3.6-10.0% of asthma patients are believed to demonstrate severe disease (14–16), which corresponds to four million patients globally. Although much less prevalent than mild and moderate asthma, severe asthma contributes to about 60% of costs associated with the disease, mainly due to higher costs of drugs and hospital care, as well as various indirect costs (17, 18). Current research efforts in the field are strongly oriented towards learning more of the pathomechanisms of severe asthma and concurrent developing of new biological therapies and identifying groups of patients best responding to a certain therapy (19). The ground-breaking change in asthma was achieved in 2003 with the first biological treatment of severe asthma: the anti-IgE monoclonal antibody omalizumab. This discovery was followed by more biological agents targeting key inflammatory nodes in the chronic inflammation underlying asthma, such as IL-5, IL-5R, IL-13 and IL-4R. Each of these drugs targets a certain immunological pathway that triggers and controls airway inflammation. Currently, omalizumab, mepolizumab, benralizumab, reslizumab, dupilumab and tezepelumab are those approved by the FDA for the treatment of severe asthma (20). With the variety of monoclonal antibodies currently available for treating asthma, clinicians may now personalize therapy according to asthma phenotype

Omalizumab is a humanized IgG1/к monoclonal antibody that binds to the IgE immunoglobulin Fc fragment (21). Thus, it inhibits the main mediator of the type I reaction pathway. By binding free IgE molecules in the circulation, it inhibits the activation of mast cells and basophils. As a result, the number of IgE receptors on the surface of these cells declines over time, which is considered to be a critical component of the drug’s clinical efficacy. Omalizumab also inhibits binding of IgE to the low-affinity IgE receptor (FcϵRI) (22),. Launched in 2003, omalizumab has been used in severe allergic asthma and, since 2014, in chronic urticaria. In 2004, omalizumab was the very first monoclonal antibody to be included in Step 5 of the GINA recommendations as an addition to standard therapy with *inter alia* high doses of inhaled steroids or β2-agonists. Since then, clinical and observational studies have found its use in improving asthma control, relieving symptoms, reducing exacerbation risk and improving lung function (23–25). The drug is known to be safe for long-term use regarding oncological safety and can be safely used during pregnancy (26–28) and by children (29).

Another biological drug in severe asthma is mepolizumab, which was registered in 2015. This antibody binds IL-5, thus preventing it from binding to the IL-5R α subunit on eosinophils. This IL-5 signal blockade reduces the eosinophil population in patients with eosinophilic asthma, leading to clinical improvement (30). Clinical and observational studies confirm that mepolizumab improves asthma control, reduces the number of exacerbations and steroid doses and improves lung function in severe eosinophilic asthma (31, 32). Importantly, both mepolizumab and omalizumab exhibit a comparable safety profile (33).

Benralizumab – registered in the US in 2017 – is a monoclonal antibody targeting IL-5R α subunit (20). Randomised clinical studies have shown the drug’s efficacy and safety in patients with severe asthma and elevated eosinophils (34, 35). It was shown to be effective in lowering exacerbation rates, symptom burden, and oral glucocorticoid use, together with improvements in lung function (36, 37). This was also confirmed in real-world studies, including 2- and 3-year-long observations (38, 39)

Another anti-IL-5 antibody is reslizumab, which was registered in the US in 2016. (anti-IL-5 antibody, US registration in 2016)

Dupilumab is a monoclonal antibody inhibiting IL-4 and IL-13 signaling. It was registered in the US in 2017) and (20, 40).

The newest drug, tezepelumab, was registered by the FDA in December 2021. It is a human, IgG2 monoclonal antibody blocking thymic stromal lymphoprotein (TSLP). This makes it a first-in-class candidate for a new group of antibodies targeting alarmins – key epithelial inflammatory cytokines involved in asthma pathogenesis (TSLP, IL-25 and IL-33). The drug has been intensively studied in recent years and promising results of phase II and III trials have been recently published (41).

The clinical efficacy of tezepelumab has been demonstrated in the following pivotal clinical studies carried out in the period 2017-2020: PATHWAY (Phase IIb), NAVIGATOR (Phase III), SOURCE (Phase III) and CASCADE3 (Phase II). Their results, published in 2021, confirm that tezepelumab is effective in a very wide population of patients with severe asthma. This has been attributed to its ability to inhibit TSLP - the mediator at the top of the inflammatory cascade.

The results of PATHWAY were published in 2017. This study was the first to examine the efficacy of tezepelumab in patients aged 18-75 with uncontrolled asthma receiving long-acting beta-agonists and medium-to-high doses of inhaled glicocorticosteroids. The drug was administered subcutaneously at three doses, *viz.* 70mg, 210mg or 280 mg, every four weeks and compared to placebo. The patients were also characterized by blood eosinophil count (<250 or ≥250), FeNO (<24 or ≥24) and Th2 status (low or high). The annualized asthma exacerbation rates at week 52 were 0.27 (70 mg), 0.20 (210mg) and 0.23 (280 mg), compared with 0.72 in the placebo group. In addition, prebronchodilator FEV1 changed by 0.12 liters, 0.13 liters and 0.15 liters at week 52 compared to baseline and was higher than in the placebo group in the three respective study groups (42). Moreover, the drug was reported to be effective in improving patient-reported quality-of-life and symptom severity compared to placebo (43). A *post hoc* analysis of the study results found that the 210mg dose reduced exacerbation rates by 64-82% across all four seasons, with the greatest reduction in summer and lowest in winter (44).

The NAVIGATOR study of tezepelumab included 1,061 patients with severe asthma. Although the study entry criteria did not include peripheral blood eosinophil counts, approximately 50% of patients were estimated to have ≥300 cells/µL. The annual rate of exacerbations in the entire study population decreased by 56% and hospitalization by 85% during tezepelumab treatment. A 70% reduction of exacerbations was noted in the population with eosinophilia ≥300 cells/µl, and 41% in the group with <300 cells/µl. Patients treated with tezepelumab achieved a 130 ml increase in FEV1 over the study, which was statistically significant. A statistically significant improvement in quality of life was also reported: the tezepelumab group demonstrated a 0.33 point better ACQ-6 score and 0.34 point better AQLQ score. In addition, the patients with eosinophilia above 300 cells/µl also demonstrated greater improvement in FEV1 and ACQ-6 and AQLQ questionnaire scores (45).

Another phase III study on tezepelumab was the SOURCE study, which aimed to assess its effectiveness in reducing the dose of oral steroids in the course of steroid-dependent asthma among 150 patients. Although no statistically significant differences were found between the study drug and placebo among patients in general, tezepelumab treatment enabled a reduction of the oral steroid dose in the population of patients with > 150 cells/µl peripheral eosinophilia (46).

A continuation of the NAVIGATOR and SOURCE studies is the ongoing DESTINATION study, in which patients will continue treatment with tezepelumab for another year or, if they were taking placebo, will be re-randomized in a 1: 1 ratio. The aim of the study is a long-term evaluation of the tolerability, safety and efficacy of tezepelumab in a cumulative two-year follow-up. The results of this study will be known soon: the planned completion date is May 2022 (47).

Tezepelumab has been recently registered by the FDA (December 2021), and European registration by the EMA was authorized in September 2022. Information on the monoclonal antibodies currently registered in severe asthma treatment may be found in **Table 1**.

**Table 1:** Current clinical options with monoclonal antibodies in severe asthma.

DRUG	FORM	TARGET	WAY OF TREATMENT, TIME INTERVAL AND DOSE	BIOLOGICAL EFFECTS	CLINICAL EFFECTS	OTHER FDA-APPROVED INDICATIONS
Omalizumab	Humanized IgG1/к, monoclonal antibody	IgE	s.c. 2 or 4 weeks interval from 75 to 600 mg depending on patient’s weight and initial total IgE	↓ circulating total IgE Down-regulation of FcϵRI receptors on basophils, mast cells, and dendritic cells	Improvement of lung function (FEV1) Improvement of quality of life (AQLQ) Improvement of asthma control (ACT) ↓ oral and inhaled corticosteroid use Reduction in exacerbation and hospitalization frequency	Chronic idiopathic urticaria
Mepolizumab	Humanized IgG1/к, monoclonal antibody	IL-5	s.c. 4 weeks interval 100 mg	Blockage of IL-5/IL-5R binding on eosinophils ↓ blood eosinophils ↓ sputum eosinophils	Reduction in exacerbation frequency *vs* placebo Improvement in AQLQ *vs* placebo No significant effect on FEV1, PEF, PC_20_	NA
Benralizumab	Humanized IgG1/к, monoclonal antibody	IL-5 Receptor alpha subunit (IL-5Rα)	s.c. 4 weeks interval (first 3 doses), then 8 weeks interval 30 mg	↓ eosinophils and basophils *via* antibody dependent cell mediated cytotoxicity (ADCC)	Reduction in exacerbation frequency No significant effect on FEV1 Mixed data on quality of life and asthma symptom scores	NA
Dupilumab	human IgG4 monoclonal antibody	IL-4 Receptor alpha subunit (IL-4Rα)	s.c. 2 weeks interval 600 mg – 1^st^ dose, then 300 mg (in adults) In children 6-11-years-old doses from 100 mg to 300 mg depending on weight and treatment interval (2 or 4 weeks)	Blockage of IL-4/IL-4Rα binding Blockage of IL-13/IL-4Rα binding	Reduced rate of severe asthma exacerbations and improved lung function (FEV1), asthma control and quality of life	Atopic dermatitis, moderate-to-severe atopic dermatitis in adolescents Chronic rhinosinusitis with nasal polyps
Reslizumab	humanized IgG4/κ mAb	IL-5	i.v. 4 weeks interval From 100 to 575 mg depending on patient’s weight	Blockage of IL-5/IL-5R binding ↓ circulating eosinophils ↓ sputum eosinophils	Reduced exacerbations, improved FEV1, forced vital capacity, the 7-item Asthma Control Questionnaire	NA
Tezepelumab	human, IgG2 monoclonal antibody	TSLP	s.c. 4 weeks interval 210 mg	Blockage of TSLP/TSLP-receptor binding	Inhibition of late allergen-induced asthmatic response (FEV1) Reduction of annualized asthma exacerbation rate	NA

NA, none available. ?, Downregulates or lowers.

### Dead ends in severe asthma research - promising, yet unsuccessful drugs in recent years

Despite the development of groundbreaking new therapies for severe asthma over the last two decades, certain groups of patients still do not respond to available therapies and hence, there still remains a substantial need for further therapeutic options. This is particularly the case among those who cannot be clearly categorized to a certain severe asthma phenotype and fail to meet the selection criteria for a particular monoclonal antibody. Moreover, even those who present as candidates for good response to a certain therapy may never be completely certain of success. Current research is also focused on search for biomarkers of possible response to available biological therapies (48).

Hence, many potential alternatives to current therapeutic strategies have recently been investigated. Some of these, discussed below, are at a late stage of research; however, they have not yet reached the assumed clinical endpoints in RCTs, and therefore have not received approval in the treatment of severe asthma. In this section we summarize the available information on drugs that were considered and studied in the area of severe asthma, yet were eventually discontinued in this indication.

## Tralokinumab

Tralokinumab is a human IgG4 monoclonal antibody targeting IL-13. The drug was studied in several clinical studies and reached phase III in STRATOS 1, STRATOS 2 and TROPOS studies. STRATOS 1 study was aimed to identify a biomarker-specific sub-group that would potentially benefit most from 300mg tralokinumab and that was further studied in STRATOS 2. The group comprised patients with baseline FeNO 37 ppb or higher, who demonstrated a reduced asthma exacerbation rate in STRATOS 1, but not in STRATOS 2 (49). The results of the TROPOS study indicate that tralokinumab use does not allow any reduction of oral corticosteroid use by patients with OCS-dependent asthma (50–52).

The phase II MESOS study examined whether tralokinumab would inhibit the release of eosinophil chemotactic factors in the lungs, resulting in decreased eosinophil lung population, despite increasing the overall eosinophil population possibly due to inhibition of eosinophil–endothelial adhesion, as observed in previous studies (53–55). The findings indicate that tralokinumab does not affect eosinophilic inflammation in bronchial submucosa, blood or sputum compared to placebo, although it did reduce FeNO and IgE concentrations (56).

A meta-analysis of six available RCTs of tralokinumab, i.e. those mentioned above with additional two phase II studies (53, 54), found that the drug improved FEV1 and FVC in patients with moderate-to-severe asthma; however, it did not improve asthma-related quality of life, nor reduce asthma exacerbations in unselected patients. Treatment has nevertheless resulted in improvements in asthma exacerbation rates in patients with high FeNO (57). Tralokinumab has been shown to be well-tolerated with a low risk of adverse events and low likelihood of immunogenicity (58).

Hence, tralokinumab treatment does not appear to be an effective strategy for severe uncontrolled asthma (59). Research has shifted from asthma to treating skin conditions, and the drug has shown promising results in atopic dermatitis (60)

## Lebrikizumab

Lebrikizumab is another humanized IgG4 monoclonal antibody targeting IL-13 that has been intensively studied in moderate-to-severe asthma. It has been evaluated in several phase II and phase III studies. In phase II studies it has demonstrated reduced exacerbation rates and improved FEV1 in patients with uncontrolled asthma, particularly among those with high periostin concentration or blood eosinophil count (61).

Replicate phase III studies - LAVOLTA1 and LAVOLTA2 - have analyzed the effects of subcutaneous lebrikizumab treatment, 37.5 mg and 125 mg once every four weeks, compared to placebo; the patients have been divided into biomarker-high (periostin, blood eosinophils) and biomarker-low subgroups. However, the results remain inconsistent, as the primary endpoint, i.e. a significant (greater than 30%) reduction of exacerbation rate, was reached in LAVOLTA1, but not in LAVOLTA2. The drug indeed improved FEV1 in the biomarker-high patients, but did not improve secondary outcomes, *viz.* AQLQ(S) and ACQ-5 scores, in either group (62).

Another pair of replicate phase III studies were LUTE and VERSE. They were primarily designed as phase III trials, but were converted to phase IIb due to the discovery of a host-cell impurity in the study drug material. Thus, the findings only included the placebo-controlled period of variable duration, and were pooled across both studies. Changes in exacerbation rate were far more pronounced in the periostin-high group (60% reduction) than in the periostin-low group (5% reduction); these two groups also demonstrated 9.1% (high group) and 2.6% changes (low group) in FEV1 (63).

Recent reports indicate that research into lebrikizumab has been moved from asthma to atopic dermatitis and chronic spontaneous urticaria. However, it may be further investigated in sub-populations of asthma patients with high blood eosinophil count and high FeNO (64).

A meta-analysis of lebrikizumab and tralokinumab studies found that although IL-13 inhibitors showed some benefits in clinical studies, a more promising approach would be the combined blocking of IL-13 and IL-4, which demonstrate overlapping pathophysiological roles (65).

## Secukinumab

Secukinumab is a human IgG1κ monoclonal antibody targeting IL-17A. It is currently registered for the treatment of plaque psoriasis, psoriatic arthritis and ankylosing spondylitis. Although the safety, tolerability and efficacy of the drug in patients with uncontrolled asthma was investigated in a Phase II study, no improvements in ACQ were found and the investigation was discontinued by the producer. This is the only available clinical study of secukinumab in severe asthma (66).

Previously, secukinumab had been evaluated in the ozone-induced airway neutrophilic inflammation model in healthy volunteers. Following ozone stimulation, study subjects were randomized to receive secukinumab (10 mg/kg), placebo or a single-dose oral corticosteroid treatment. No significant differences in airway neutrophilia compared to baseline were observed between study groups, including the secukinumab group (67). These findings suggest that the drug would probably not bring any clinical improvement in neutrophilic asthma.

## Brodalumab

Brodalumab is a human, IgG2 monoclonal antibody targeting IL-17RA, which is currently registered for the treatment of psoriasis vulgaris, psoriatic arthritis, pustular psoriasis and psoriatic erythroderma. The drug was studied in a randomized, double-blind phase II study with 315 participants in four groups: placebo, brodalumab 140 mg, brodalumab 210 mg and brodalumab 280 mg. No clinically significant differences were observed between the groups in terms of ACQ score, FEV1, morning PEF, SABA use, daily and nighttime symptom scores or symptom-free days. A predefined subgroup analysis found that only the high bronchodilator reversibility subgroup demonstrated clinically significant benefits (68).

Another phase II study of brodalumab with 421 patients was initiated but later terminated; however, this was due to lack of observed efficacy, not safety concerns. The results are not publicly available (69).

## Fevipiprant

Another mediator pivotal to orchestrating immunological and inflammatory mechanisms in asthma is prostaglandin D_2_ (PGD_2_), an eicosanoid which is released by degranulating mast cells. Fevipiprant, an oral, nonsteroidal, highly-selective, reversible antagonist of the DP2 receptor showed promising results in three phase II studies. Although it is not a monoclonal antibody, due to its advanced stage of development, we decided to include it in this review to broaden the context of current advances in asthma.

Early-phase trials have confirmed its safety and demonstrated its potential efficacy in patients with asthma, specifically, improvement in FEV1 and eosinophilic airway inflammation (70, 71). Despite these results, the subsequent phase III trials did not yield satisfactory clinical conclusions.

In two placebo-controlled replicate phase III studies named ZEAL-1 and ZEAL-2, patients received 150 mg fevipirant (or placebo) plus standard-of-care asthma therapy (medium- or high-dose ICS, low-dose ICS plus either LABA or LTRA, or medium-dose ICS plus LABA for at least three months prior to screening). Neither study met its primary endpoint, defined as change from baseline in pre-dose pre-bronchodilator FEV1 at the end of the 12-week treatment period. Nor did it meet its secondary endpoints: change from baseline in daytime asthma symptom score and total dailySABA use over 12 weeks of treatment, and change from baseline in AQLQ +12 score at week 12 (72).

Another two replicate phase 3 studies of fevipirant (LUSTER-1 and LUSTER-2) examined the effects of fevipiprant on moderate to severe asthma exacerbation annualized rate in patients aged 12 or older receiving GINA step 4 or 5 treatment. Patients were randomised to receive 150 or 450 mg of fevipirant or placebo throughout a 52-week observation period. Neither of the studies demonstrated a significant reduction in asthma exacerbation annualized rates (73). Due to this observed overall lack of clinical efficacy, the manufacturer has discontinued its research in asthma.

## Anakinra

Anakinra is a human IL-1 receptor antagonist produced by recombinant DNA technology in an *E. coli* expression system. As the IL-1-regulated pathway is believed to play a significant role in asthma pathogenesis in both Th2/Th17-high and –low phenotypes, it has become an attractive therapeutic target (74). However, two recent clinical trials that were designed to assess the effectiveness of anakinra as a rescue treatment for airway inflammation in allergic asthma, either through early- or late-phase administration after allergen challenge, were withdrawn due to the COVID-19 pandemic and the risks associated with allergen exposure and anakinra treatment (75, 76). Another study of the drug has been suspended (77).

Other potential treatments for severe asthma have unfortunately failed to demonstrate satisfactory results and thus are not being continuously studied. Such examples include:

• Cendakimab (formerly known as RPC4046), a monoclonal antibody targeting IL-13, which was only reported in a Phase I study in asthmatic patients (78). The drug is being further developed in eosinophilic esophagitis (79)
• GSK 679586 – another monoclonal antibody targeting IL-13 reported to reach phase II. The drug did not demonstrate any clinically-relevant improvements in asthma control, pulmonary function or exacerbations in patients with severe asthma (80).
• Daclizumab - a monoclonal antibody that binds the IL-2R α chain (CD25), which in turn inhibits lymphocyte activation. The available literature only includes one RCT for the drug: A 2008 study by Busse et al. examined its effects on FEV1 changes in 115 patients with moderate to severe uncontrolled asthma. An improvement was observed in the 88-patient daclizumab group (4.4 ± 1.80% *vs* 1.5 ± 2.39%; p = 0.05), daytime asthma symptoms were reduced (p = 0.018), and the time to exacerbation was prolonged (p = 0.024). An absolute increase of FEV1 was observed in the treated group, i.e. from 2.34 ± 0.07 in baseline to 2.4 ± 0.08 at Day 84, the patients receiving placebo had a decrease in FEV1 (from 2.25 ± 0.1 to 2.2 ± 0.1 L), and an increase in serious adverse events was reported in the treatment arm (5 *vs* 1) (81). Although it was discussed at the time as a potential asthma therapy, the drug was not further studied in asthma (82). Daclizumab is now registered and indicated in multiple sclerosis (83)
• Enokizumab/Medi-528 - an antibody targeting IL-9: an inflammatory cytokine that regulates the development of airway inflammation, mucus production, airway hyperresponsiveness, and airway fibrosis by increasing mast cell numbers and activity (84). Enokizumab showed an acceptable safety profile in phase I studies (85). The drug reached Phase II clinical trials in 2011 (86). However, at this stage, the drug administration (dosed subcutaneously at three dosages – 30, 100 and 300 mg every 2 weeks for 24 weeks in addition to concurrent asthma medication) did not yield any improvements in ACQ-6 scores, asthma exacerbation rates or FEV1 values (87).
• Canakimumab – a monoclonal antibody targeted at IL-1β. The drug was tested in one small study in 16 asthmatics with positive results, with attenuation of the late asthmatic response after inhalative allergen challenge and a >90% decrease of IL-1β level (88). The drug is currently registered in Periodic Fever Syndrome, Cryopyrin-Associated Periodic Syndromes, Tumor Necrosis Factor Receptor-Associated Periodic Syndrome, hyperimmunoglobulin D syndrome and several other rheumatoid diseases.
• Risankizumab - a monoclonal antibody targeting the p19 subunit of IL-23: a cytokine mostly affecting Th17 cells and thus decreasing antigen-induced Th2 cytokine production (89). Only one phase IIa study on this drug has been published to date. The results indicate that treatment failed to reduce the annualized asthma exacerbation rate compared to placebo, with the risankizumab group demonstrating shorter time to first asthma worsening (90). Therefore, research in this area is discontinued, with potential other applications (e.g. psoriasis) being currently researched (91).
• VR942 - a dry-powder formulation containing CDP7766, a high-affinity anti-human-IL-13 antigen-binding antibody fragment. Only one phase I study has examined the safety and pharmacodynamics of the drug. Although the concept of direct inhalable administration of monoclonal antibodies to the target tissue seems promising, this study remains one of few such reports in humans (92). A 2012 study by Hacha et al. examined the concept of nebulized anti-IL-13 antibody treatment in a murine model of asthma with promising results; however, the idea was not continued (93). Even so, other studies have evaluated the potential of similar drug delivery methods in other respiratory conditions (94).

A summary on the above-mentioned discontinued drugs in severe asthma is presented in **Table 2**.

**Table 2:** Drugs discontinued in severe asthma research.

DRUG	FORM	TARGET	LAST PHASE OF STUDY	LAST PHASE OF STUDY YEAR	OTHER FDA-APPROVED INDICATIONS
Tralokinumab	Human IgG4 monoclonal antibody	IL-13	Phase III	2019 (51)	Atopic dermatitis
Lebrikizumab	Humanized IgG4 monoclonal antibody	IL-13	Phase III	2016 (62)	NA, possibly atopic dermatitis
Secukinumab	Human IgG1κ monoclonal antibody	IL-17A	Phase II	2015 (66)	Plaque psoriasis, Psoriatic arthritis, Ankylosing spondylitis
Brodalumab	Human, IgG2 monoclonal antibody	IL-17RA	Phase II	2013 (95)	Plaque psoriasis
Fevipirant	Non-steroidal selective receptor inhibitor	CRTH2	Phase III	2021 (72)	NA
Anakinra	Protein	IL-1R	Phase I/Phase II		Rheumatoid arthritis, COVID-19, Periodic fever syndrome, Cryopyrin-associated periodic syndromes, Familial Mediterranean Fever, Adult-onset Still’s disease
Cendakimab	Monoclonal antibody	IL-13	Phase I	2017 (78)	NA
GSK 679586	Monoclonal antibody	IL-13	Phase II	2014 (80)	NA
Daclizumab	Humanized IgG1 Monoclonal antibody	IL-2Rα	Phase II	2008 (81)	Multiple sclerosis
Enokizumab/ Medi-528	Humanized IgG1κ monoclonal antibody	IL-9	Phase II	2013 (87)	NA
Canakimumab	Humanized IgG1κ monoclonal antibody	IL-1β	Phase I/Phase II	2006 (88)	Relapsing fever syndrome, Cryopyrin-Associated Periodic Syndromes, Tumor Necrosis Factor Receptor-Associated Periodic Syndrome, hyperimmunoglobulin D syndrome and several other rheumathoid diseases
Risankizumab	Human IgG1 monoclonal antibody	IL-23	Phase IIa	2021 (90)	Plaque psoriasis Psoriatic arthritis
VR942	Inhalable fragment of antibody	IL-13	Phase I	2018 (92)	NA
Itepekimab (REGN-3500)	Human IgG4P monoclonal antibody	IL-33	Phase II	2021 (96)	NA
Etokimab	Monoclonal antibody	IL-33	Phase IIa	2019 (97)	NA
Melrilimab	Monoclonal antibody	IL-33	Phase II	2020 (98)	NA
LY3375880	Monoclonal antibody	IL-33	Preliminary	2020 (99)	NA

NA, none available.

### Other approaches for treating severe asthma and potential new targets

Research on monoclonal antibodies in severe asthma has made significant progress in recent years. With the six monoclonal antibodies currently available (omalizumab, mepolizumab, benralizumab, reslizumab dupilumab and tezepelumab), patients with severe asthma have a fairly wide range of possible treatments. However, although these are highly-advanced drugs that target specific pathophysiological pathways of asthma, a large group of patients still fails to respond to treatment (100). Consequently, the terms ‘non-responders’, ‘responders’ and ‘super-responders’ have emerged to categorize those who do or do not reach improvements with biologics. This problem may be partially explained by the fact that the target molecule is a part of a causal network of many other inflammatory mediators rather than an element of a linear cause and effect relation (101). Many efforts have been made to identify biomarkers of response to biological treatment, yet no dichotomous factor has been found to date (101–104).

As such, research has turned to new antibodies aimed at other cytokines. This section summarizes the current findings concerning molecular targets in severe asthma obtained in clinical and pre-clinical research.

### Tocilizumab (also called ‘atlizumab’)

Tocilizumab is a humanized IgG1 antibody targeting IL-6R – the receptor for IL-6, that is an interleukin recently attributed to contribute to asthma pathogenesis and which may represent a pathophysiological target (105). This drug is registered for the treatment of rheumatological conditions such as rheumatoid arthritis. More recently, it has been intensively studied and registered as a drug in SARS-COV-2 infection (106). However, as of May 2022, there have been no clinical studies of tocilizumab in severe asthma, and very limited data exists on the use of the drug in asthma.

In May 2019 Esty et al. reported two pediatric cases of severe persistent, non-atopic asthma treated with tocilizumab. Both patients demonstrated good clinical (FEV1 increase, reduction of oral corticosteroids) and immunological (reduced IL-4 and IL-17 production) responses to the therapy and no adverse events (107).

A proof-of-concept study by Revez et al. published in June 2019 studied the effects of tocilizumab on asthma patients with high sIL-6R levels following two allergen inhalation challenge tests. The study included 11 patients: six who received tocilizumab and five placebo. No significant differences in the primary endpoint was observed between study arms: late asthmatic response, maximum percentage fall in FEV1 and AUC of the percent fall in FEV1 (108).

## Anti-IL-33: Itepekimab, etokimab, melrilimab, LY3375880, MEDI3506

Itepekimab, found in the literature under the aliases REGN-3500 or SAR440340, is an anti-IL33 antibody that has recently been studied or treating asthma. Although research on this antibody has reached phase II, the results are insufficient to continue it further. A recent study by Weschler et al. compared the efficacy of itepekimab with three other treatments: dupilumab, itepekimab+dupilumab and placebo. The groups were randomized in a 1:1:1:1 ratio and the primary endpoint was the occurrence of an event indicating loss of asthma control. By week 12, such an event occurred in 22% of patients in the itepekimab group, 27% in the combination group, and 19% in the dupilumab group, as compared to 41% in the placebo group (96). As a consequence of these results, the drug was discontinued for asthma research in February 2021; however, its potential against COPD remains under study (109).

Etokimab is another anti-IL-33 monoclonal antibody which has been recently studied as a possible asthma treatment. Currently, no peer-reviewed reports are available; however, it has been speculated that the drug might be studied against asthma, pending results of failing studies in eczema and atopic dermatitis (99).

Other anti-IL-33/IL-33R drugs reported are melrilimab by GSK, LY3375880 by Lilly and MEDI3506 by AstraZeneca. Only the latter is currently being studied in an on-going phase II clinical trial, the FRONTIER-3 trial, with an estimated completion date in August 2022 (110).

## Bispecific antibodies

An interesting approach, although still only in the initial research phase and without any significant progress regarding severe asthma biological therapy, is based on the concept of bispecific antibodies, i.e. such that one particle can target two immunological targets at the same time. Compared with hypothetical combination therapy including two monospecific antibodies, bispecific antibody treatment may incur lower costs of development and clinical trials. Such examples include:

• BITS7201A (a monoclonal antibody that binds both IL-13 and IL-17) which to date has only been studied in a single phase I study; it showed good drug tolerance, but a high incidence of anti-drug antibodies (111)
• a monoclonal antibody simultaneously targeting IL-4Rα and IL-5 in a murine model of asthma (112)
• bispecific anti-TSLP/IL13 antibodies called Zweimabs (monovalent bispecific) and Doppelmabs (bivalent bispecific) (113)

**Figure 1:**
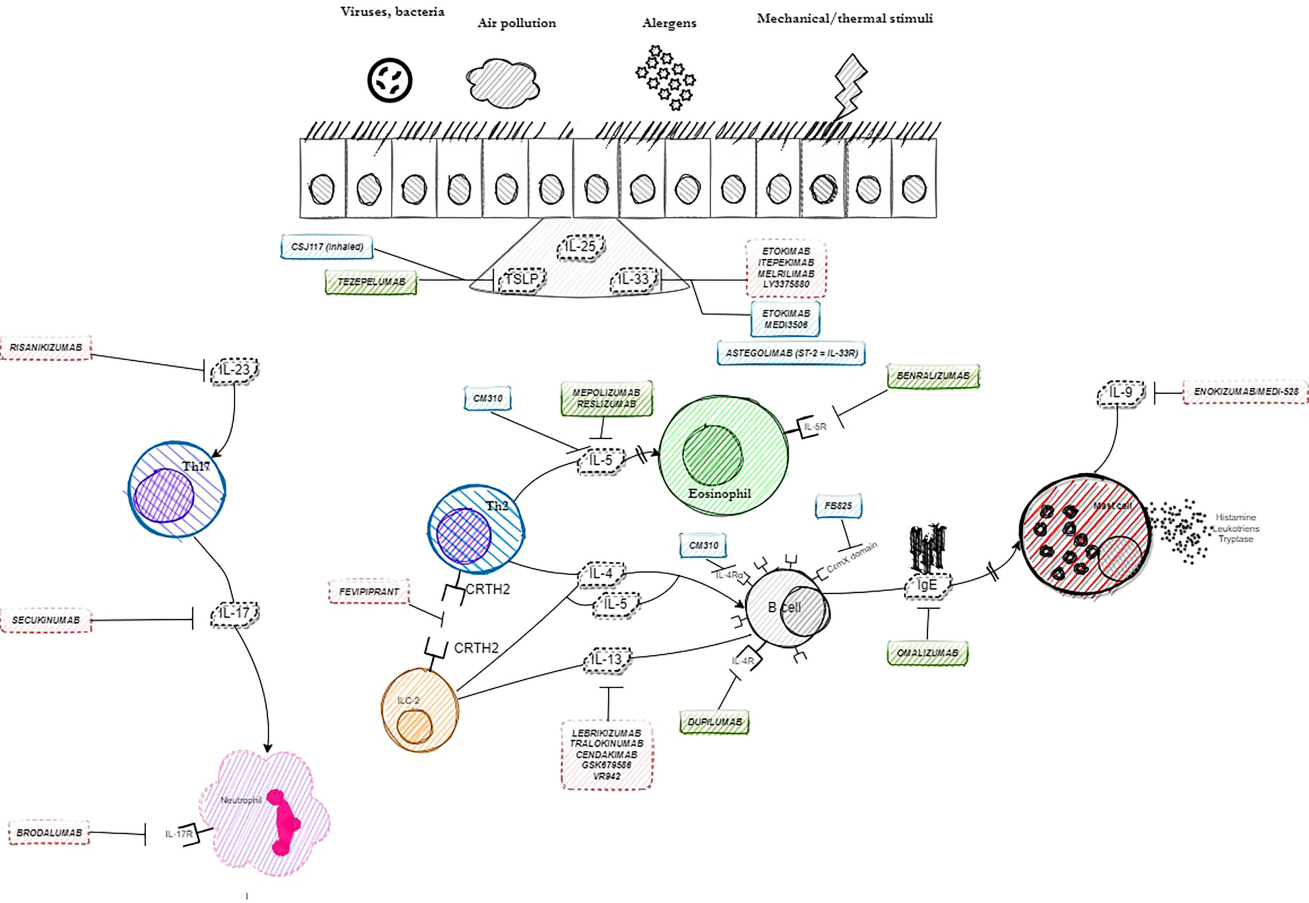
Summary on available, continued and discontinued agents in severe asthma.

**Figure 2:**
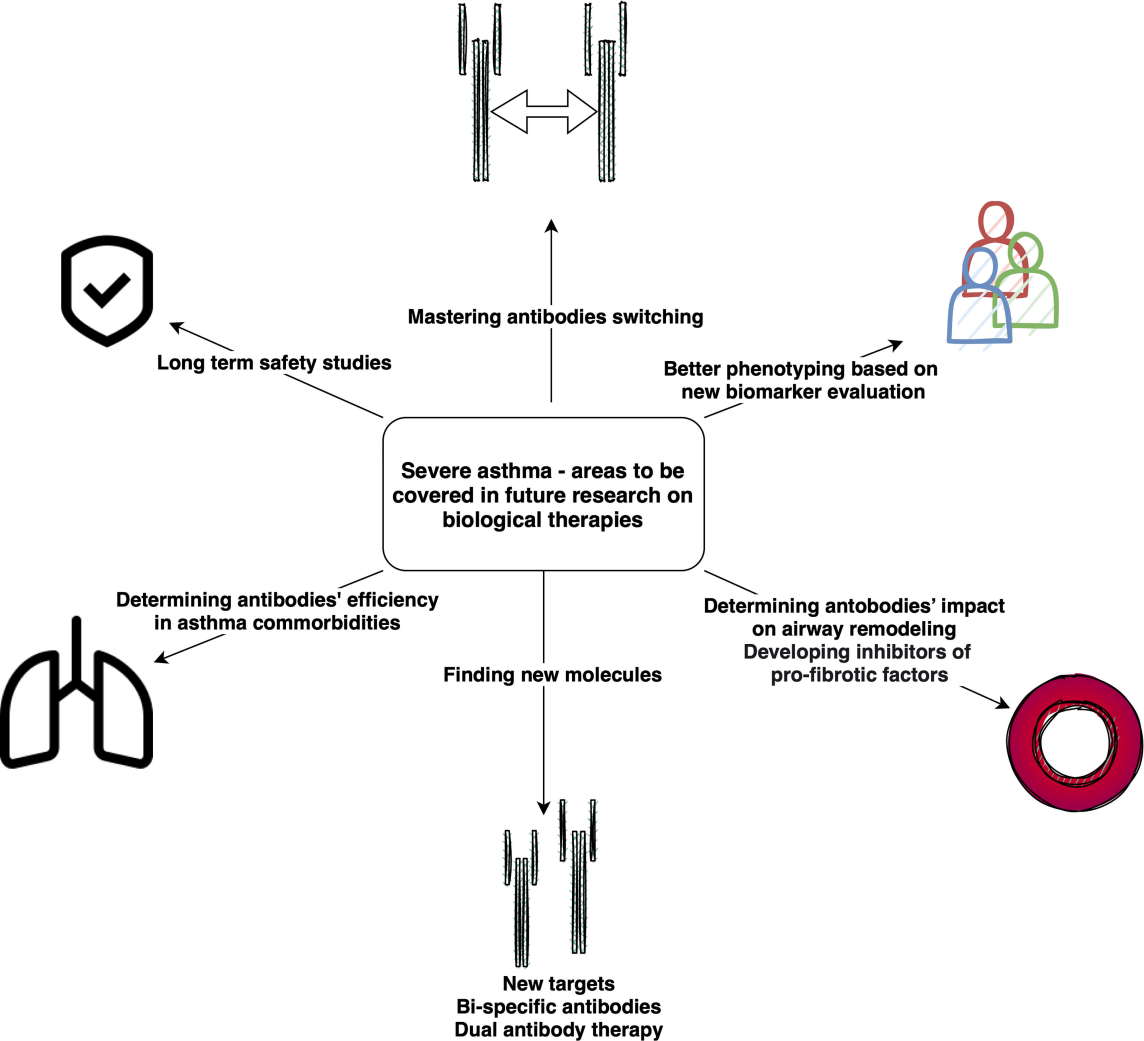
Summary on future perspectives in severe asthma research.

Selected other particles with signaled or ongoing research in severe asthma are presented in **Table 3**. The summary on available, currently researched and discontinued agents in severe asthma is shown in **Figure 1**. Also, a brief graphic summary on future perspectives of research in the field of severe asthma may be found in **Figure 2**.

**Table 3:** Monoclonal antibodies and drugs in current clinical trials or suggested as potential agents in severe asthma.

DRUG	FORM	TARGET	CURRENT STUDY STAGE	OTHER FDA-APPROVED INDICATIONS
Tocizilumab	Humanised IgG1 monoclonal antibody	IL-6R	Casuistic	Rheumatoid arthritis COVID-19 Juvenile idiopathic arthritis Cytokine release syndrome
Clazakizumab	Humanized monoclonal IgG1 antibody	IL-6	Phase 2 (114)	NA
CSJ117	Antibody fragment in powder for delivery to the lungs *via dry powder in*haler	TSLP	Phase 2 (115)	NA
FB825	Humanised IgG1 monoclonal antibody	CϵmX domain on human B lymphocytic cells expressing membrane-bound IgE (mIgE).	Phase 2 (116)	NA
CM310	Monoclonal antibody	IL-4Rα	Phase 2 (117)	NA
610	Monoclonal antibody	IL-5	Phase 1 (118)	NA
FB704A	Human antibody	IL-6	Phase 2 (119)	NA
MEDI3506	Monoclonal antibody	IL-33	Phase 2 (120)	NA

NA, none available.

### Challenges and future perspectives in the biological therapy of severe asthma

Clearly, research of new drugs for severe asthma remains intense, with many potential pathways under investigation. Parallel to the development of new drugs, attempts are being made to optimize the use of existing therapies and to better understand their mechanisms. One promising direction involves identifying the biomarkers of response to a specific monoclonal antibody, an important aspect of personalizing treatment, while another concerns improving the phenotyping of asthma, and thus the selection of a drug compatible with the immune background. For example, many patients qualify for both omalizumab (high IgE) and anti-IL-5 therapies (high blood eosinophilia), forcing a difficult decision in the choice of a drug (121), which may be suboptimal (122).

However, as few studies have compared individual molecules under real-life and head-to-head conditions, there is often inadequate data to conclusively state that a given drug is better than another. As such, the choice of treatment remains a clinical challenge, not only due to the differentiation of the disease, but also to the variety of potential treatment options (123). Although some ongoing head-to-head studies cover this issue, such as PREDICTUMAB, a head-to-head study of omalizumab and mepolizumab (124), or Choosebetweenmab (125), they are few in number. Notably, no direct head-to-head comparisons between anti-IL-5 antibodies in asthma have been made, although one head-to-head study comparing benralizumab and mepolizumab in Eosinophilic Granulomatosis With Polyangiitis is ongoing (MANDARA) (126). Therefore, no clear advantage can be found for any of these treatments (127, 128).

Another important aspect of research into severe asthma and implementation of new drugs concerns their effect on airway remodeling: an important aspect of research, with great efforts being aimed at determining whether new, or existing, drugs reverse this process. For example, the effect of tezepelumab on bronchial remodeling was already verified in phase II studies (129), unlike the majority of previously registered drugs (130).

Finally, no matter how successful the new and future biologics may be at treating severe asthma, research is still needed into the selection of an appropriate treatment depending on the individual characteristics of the patient’s disease. There is a clear need for updating state-of-art algorithms biologics selection to allow them to reflect nuances in asthma phenotype and treatment response. Some progress in this field has been made recently with algorithms proposed by Papadopoulos (131), Viswanathan (123) or Buhl (132).

## Summary

Monoclonal antibodies targeting specific inflammatory cytokines are undoubtedly revolutionary drugs in many fields of medicine and have begun a new chapter in the treatment of severe and complex cases of immunological diseases. This is also the case in severe asthma, where we have moved from demanding and aggravating oral steroid therapy to a targeted and personalized immunological approach. In asthma, the use of monoclonal antibodies has given many patients the chance to control their disease and significantly improve their quality of life. However, there is still a need to develop new therapies that will be effective in more complex and unusual cases, or where existing treatment has not been successful.

Research on new monoclonal antibodies in asthma does not always bear fruit. The immunological complexity of the disease, with its considerable variation in phenotypes and endotypes, greatly hinders the identification of new therapeutical solutions. The bench-to-bed process of drug development is always a challenge which continually demands greater efforts and many of the promising concepts are not confirmed in clinical trials; however, while this may appear as failure, these findings allow future research in a given area to be narrowed or redirected to other areas of medicine.

## Author contributions

GK, PD, MP and MK created the concept of the paper. GK conducted the literature research and wrote the initial version of the manuscript. PK, MP and MK revised the paper. All authors contributed to the article and approved the submitted version.
